# Historical issues of hydrotherapy in thermal–mineral springs of the Hellenic world

**DOI:** 10.1007/s40899-022-00802-1

**Published:** 2022-12-21

**Authors:** K. Voudouris, C. Yapijakis, Μ.-Ν. Georgaki, A. N. Angelakis

**Affiliations:** 1 Laboratory of Engineering Geology and Hydrogeology, School of Geology, Aristotle University of Thessaloniki (AUTh), 54124 Thessaloniki, Greece; 2 UNESCO Center for Integrated and Multidisciplinary Water Resources Management, AUTh, Thessaloniki, Greece; 3 1st Department of Pediatrics, School of Medicine, National Kapodistrian University of Athens, Aghia Sophia” Children’sHospital, Thivon 1, 11527 Athens, Greece; 4 University Research Institute of Maternal and Child Health and Precision Medicine, Athens, Greece; 5 HAO-Demeter, Agricultural Research Institution of Crete, 71300 Iraklion, Greece; 6 Union of Water Supply and Sewerage Enterprises, 41222 Larissa, Greece

**Keywords:** Asclepieia, Balneotherapy, Classical and Hellenistic periods, Medicine, Water

## Abstract

Many springs have been recorded in Greece; some of them are characterized as thermo-mineral springs and are associated with their position between Eurasia and Africa, the volcanic activity, and the presence of tectonic faults. The therapeutic use of water (hydrotherapy) has been recorded in ancient Greece since at least 1000 BC. Asclepius was the god of medicine in ancient Greek religion and priests operated his worship centers (*Asclepieia*) offering medical services in areas with proper climatic conditions. In historical times, Hippocrates from the Aegean island of Kos (460–375 BC) is considered the father of scientific medicine as well as hydrotherapy. During the Hellenistic period, the significance of water in health was widely recognized. In the Roman era, many doctors evolved hydrotherapy treatment and the use of hot baths continued in the early Byzantine period until the sixth century AD. Finally, during the Ottoman period, the kind of respiratory bath, named *Hamam*, was the dominant form in public baths. Their temperature ranges between 20.5 and 83 °C, and the dominant hydrochemical type is Na-Cl. This review describes the history of hydrotherapy in Greece through the centuries, the physicochemical characteristics of thermal springs, as well as contemporary and future trends and challenges are presented.

## Introduction

The majority of towns in ancient Greece were set up on hills close to springs, especially in the driest areas. The ancient Greeks, unlike other peoples, avoided living near rivers and lakes to be protected from water-related diseases, as well as from floods (Angelakis et al. 2020b). So, the springs (in combination with shallow wells) have been the main source of water supply in ancient Greece since the dawn of its history (Voudouris 2012). For this reason, sophisticated works were constructed to collect and transfer water from springs to towns, e.g., tunnels, cisterns, galleries, aqueducts, qanats, and fountains (Angelakis et al. 2007). Furthermore, water treatment technologies were developed in Hellenic world since early Minoan times (Crouch 2003; Angelakis et al. 2020b).

During the Homeric era (ninth century BC), warm baths used for body cleanliness and the alleviation of pain. Hippocrates (460–377 BC) wrote the first medical observations and recommendations in his book on Air, Water, and Places giving emphasis on the role of water. During the Roman period, many doctors evolved hydrotherapy treatment, with Asclepiades of Bithynia being the most prominent among them. The Roman tradition of the thermal baths flourished especially during the period of the emperor Traianus, and continued until the sixth century AD in the early Byzantine period. Nevertheless, the rise of Christianity in the Middle Ages gradually led the bathing culture into disrepute. The use of baths was officially prohibited, since faith in cure through praying was considered more important than a medicinal bath (Routh et al. 1996; van Tubergen and van der Linden 2002). Finally, during the Ottoman period, the kind of respiratory bath, named *Hamam*, was the dominant element in public baths until the modern era. Nowadays, hot springs are the basic pillars that contribute to the development of medical tourism and/or alternate tourism.

The therapy of diseases with thermal–mineral water (hydrotherapy) was one of the oldest medical treatments. Sacred centers, named *Asclepieia,* were dedicated to the healer-god Asclepius (Chaviara-Karahaliou 1984). These centers, except of sanctuaries, were operated by priests as centers offering medical advice and services, prognosis, and healing in all over Greece (Martin and Metzger 1976; Kallegia 2000). Approximately, 400 *Asclepieia* are recorded in ancient Greece operated since the early classical period, ca. fifth century BC (Angelakis et al. 2020a). *Asclepieia* were located in beautiful areas with good and dry climatic conditions and fed mainly by spring-water (Risse 1999). In all *Asclepieia*, the role of water is very important and there are no *Aclepieia* without a water source (mainly springs or rainwater by harvesting systems). In addition, baths and fountains were a basic and necessary infrastructure in any *Asclepieion.*

Later during the classical period, Hippocrates (460–437 BC) was the father of observation-based clinical medicine and hydrotherapy, considering the nature and environment fundamental elements for human health (Yapijakis 2009). In view of the above, it is concluded that there was a strong relationship between thermal springs, cleanliness, and medicine. The main uses of thermal waters include hydrotherapy, greenhouse heating, drying of agricultural products, and central heating. It is pointed out that many thermal springs in Greece still operate today for therapeutic purposes and recreation areas (see subsection 5).

Greece is located within the boundaries of the conflict between the lithospheric plates of Europe and Africa (Papachristou et al. 2014). Its position favors the occurrence of geothermal fields and thermal springs. A large part of Greece is covered by well-karstifiable carbonate rocks (limestones, dolomites, marbles), in which karst aquifers are developed (Voudouris 2019). The karst aquifers discharge through different types of springs: submarine, coastal brackish, inland freshwater, and thermal springs. Nowadays, freshwater springs contribute to the domestic water supply in many cities of Greece. Some springs are characterized as thermo-minerals or thermo-metallics, relating to the fault structures. The thermal springs of Greece are due to volcanic activity, geology, and intensive tectonic stress of Greek territory (Lambrakis and Kallergis 2005). Furthermore, some springs were not just a water source but also became a place of medical services, as well as cultural symbol on the history, related to local religion (Voudouris et al. 2019). Except of thermal springs, in many geothermal fields, boreholes have been drilled, in which hot water overflows due to artesian phenomena.

This article deals with a synoptic review of the role of mineral thermal springs in hydrotherapy in Greece through the centuries. Initially, the bibliography concerning the history of hydrotherapy is briefly presented. Also, the physicochemical characteristics, the hydrochemical water types, as well as the therapeutic effects of thermal springs in Greece are presented. Finally, contemporary and future trends and challenges in the evolution of hydrotherapy in the world, as well as in Greece are examined.

## Characteristics of thermal and mineral waters

Geothermal waters have been used for therapeutic purposes in Greece most probably since the dawn of human civilization. As mentioned before, there are many thermal springs in Greece due to geological conditions and tectonic structure that favor the circulation of fluids and the development of springs. The geotectonic regime and the presence of active faults favor the circulation of geothermal fluids and the development of thermal springs, as well as geothermal fields (Papachristou et al. 2014). Numerous thermal springs are located in Greek regions with high values of heat flow (70–100 mW/cm^2^), originating from recent volcanic and tectonic activities (Fytikas 1978). The presence of faults plays an important role in allowing the thermal water to rise from great depths to the upper aquifers. In these areas, the water of atmospheric origin, mixed with seawater (common in coastal areas and islands) or water of magma (rarely), infiltrates to great depths, is heated, becoming lighter, and then rises up along tectonic faults. The most thermal springs are recorded in Central and Northern Greece. South Aegean region includes many islands and represents an active zone between Eurasian and African plates, named south Aegean active volcanic arc.

Thermal–mineral waters are considered hot waters (> 20 °C) with total dissolved salts (TDS) concentration higher than 1 g/L, which have a beneficial physiological effect on human health, recognized by Greek laws. The thermal–mineral waters are classified into different sub-classes according to temperature, the concentration of total dissolved salts, and trace elements. In terms of temperature, the waters are grouped in classes: cold (< 20 °C), hypothermal (20–34 °C), homeothermal (35–38 °C), and hyperthermal waters with a temperature greater than 38 °C. Homeo cames from the Greek word ὃμοιος, which means the same; temperature similar to the human body. Although rare, there are cold (< 20 °C) mineral waters in Greece, rich in metallic elements, suitable for hydrotherapy (Κivotos at Grevena, Kokkino Nero at Larissa, etc.). Some of them are utilized for hydrotherapy after artificial heating and others for bottling of potable water and soft drinks (Souroti, Ksino Nero Florinas, Sariza, etc.). From the hydrogeological point of view, all groundwaters with temperatures higher than the average annual temperature of a region could be considered as thermals, albeit they cannot be used in hydrotherapy.

**Fig. 1:**
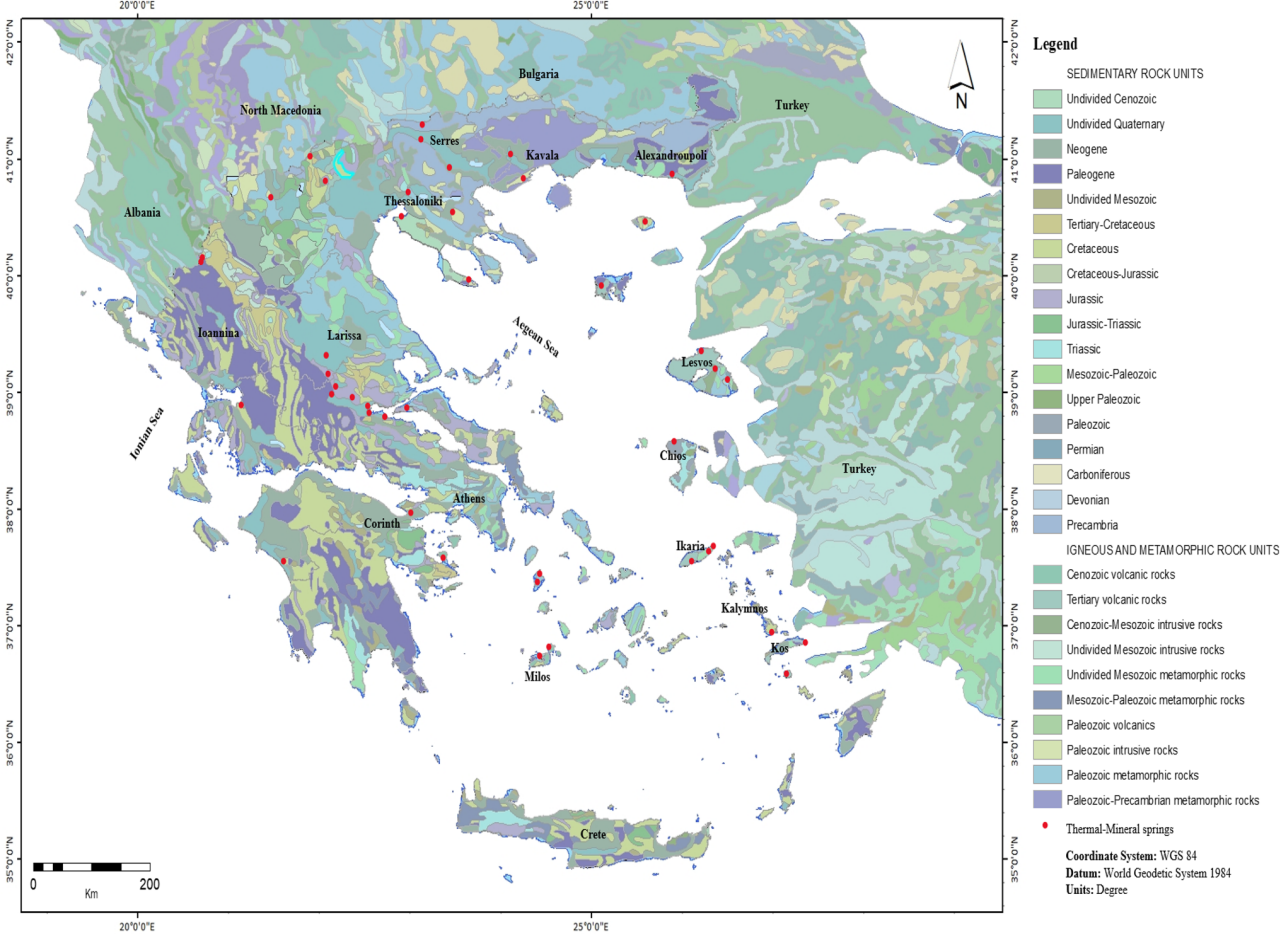
Map of Greece showing the location of the main thermal–mineral springs, certified by the Ministry of Tourism

Many scientists were studying the origin and the chemical and hydrogeological regime of springs. The systematic recording of thermal springs started after first decades of the establishment of the Greek state. Kouzis wrote in 1916 the first bibliography concerning the Greek thermal springs, which was completed by Emmanuel in 1927 (Platakis 1966). In 1938, an extensive bibliography was published by Lekkas (1938) and the existence of 750 metallic springs was mentioned. Finally, a detailed bibliography of the thermal springs of Greece was published by Platakis in 1966. Detailed data on hydrogeological and hydrochemical characteristics, as well as the origin of thermal springs of Greece, are reported by Lambrakis and Kallergis (2005). The Institute of Geology and Mineral Exploration (nowadays H.S.G.M.E.) is the official service of the state for the recording of thermal springs. According to the Hellenic Survey of Geology and Mineral Exploration (H.S.G.M.E.), there are 750 thermal springs in Greece; 128 of them are active, and 52 are recognized and certified by the Ministry of Tourism. In Appendix, the certified springs of Greece with their general characteristics are presented. The location of thermal–mineral springs, certified by the Ministry of Tourism, is shown in Fig. 1. Their hydrochemical characteristics are provided by H.S.G.M.E. (2009). The statistical parameters of the main ions are shown in Table 1.

**Table 1:** Major ions’ chemistry (in mg/L) of thermal–mineral springs

	Min	Max	Average	St. dev
Na^+^	6.9	12,452.9	3407.9	4131.7
Ca^2+^	2.0	1719.2	453.8	511.0
Mg^2+^	0.7	1447.4	274.6	379.1
Cl^−^	7.9	21,757.5	6069.5	7408.7
SO_4_^2−^	17.1	4697.7	907.6	1255.2
HCO_3_^−^	40.2	1459.3	397.4	326.6
pH	7.2	9.8	7.7	0.6
TDS	206	42,667	11,461	13,674

**Fig. 2:**
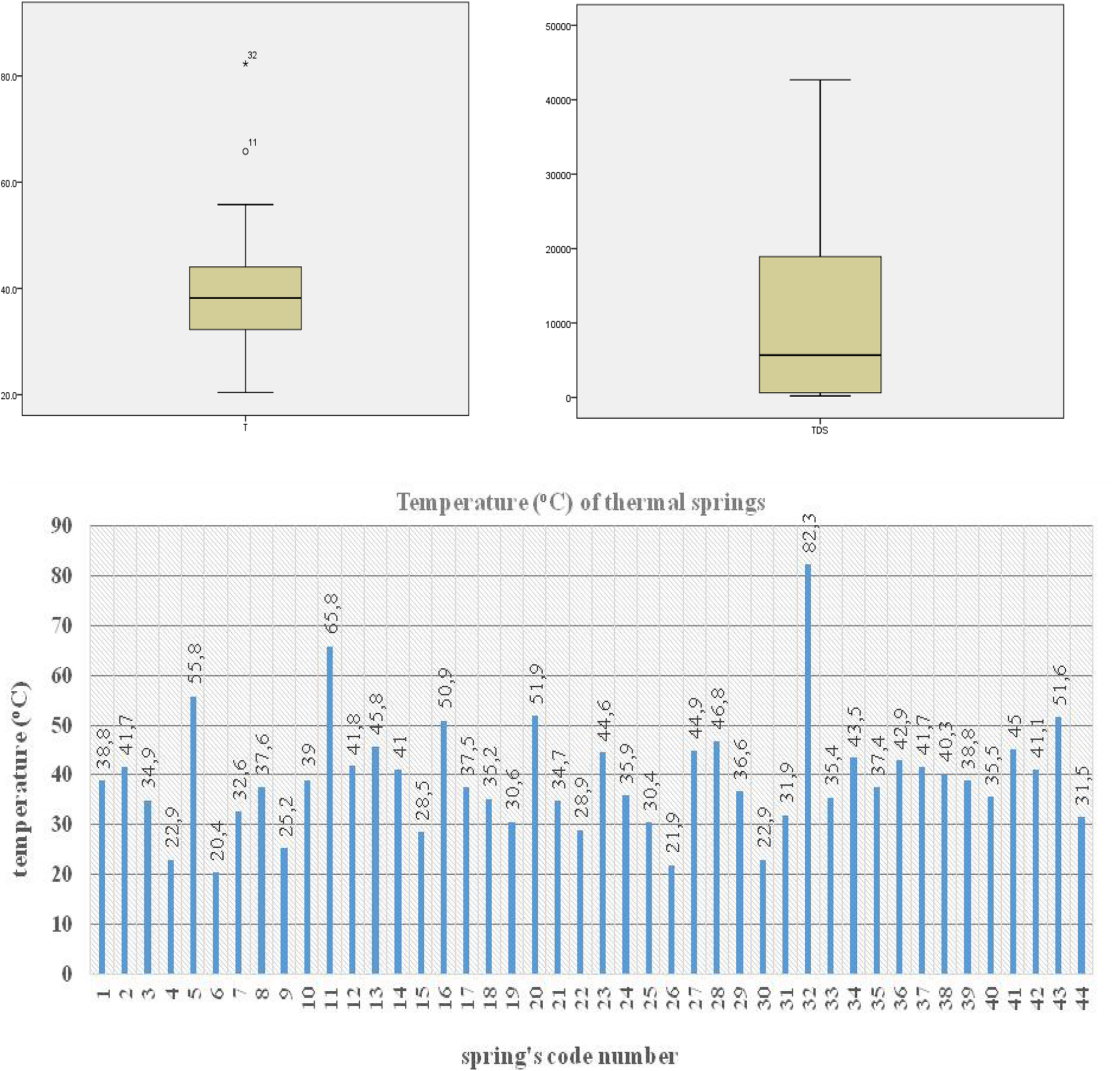
Box plot of temperature (^o^C) and TDS (mg/L) (above); fluctuation of temperature of spring-water (^o^C) (below)

The water temperature (T in ^o^C) of all discovered hydrothermal springs ranges between 20.4 °C (code number 6 in Table 1) and 82.3 °C (c.n. 32) (Fig. 2). From the boxplot of Fig. 2, it is concluded that the median value of all the springs is 38.2 °C and Q_3_ (75th percentile) = 44.32 °C, which means that 75% of the total number of springs is lower than 44.3 °C. The numbers 32 and 11 represent the outliers’ values (very high temperatures). The highest temperature (82.3 °C) value is recorded in Lesvos Island of Northern Aegean Sea. Besides, high temperatures in spring-water are recorded in Southern Aegean active volcanic arc (Kimolos, Kythnos, Milos, and Chios). Furthermore, these waters are characterized by the presence of high CO_2_ concentration, originating from deep lithogenic sources (D’Alessandro et al. 2010). High silica (SiO_2_) concentrations are locally recorded in thermal waters from Milos and Nisyros islands.

**Fig. 3:**
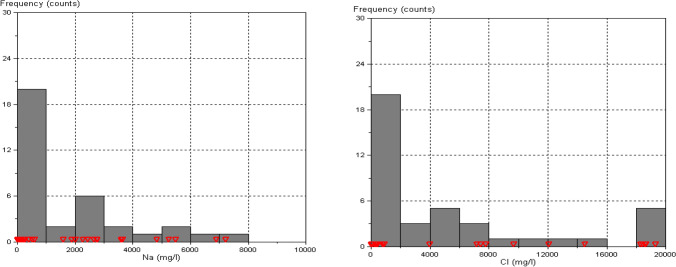
Histograms of Na and Cl in mg/L

Power of Hydrogen (pH) values range between 7.2 and 9.8 (mean value 7.7), indicating an alkaline behavior. Total Dissolved Salts (TDS) values vary between 206 and 42,667 mg/L with a mean value 11,460 mg/L and standard deviation 13,670 (Fig. 2). The dominant cation is sodium (Na) and the dominant anion is chloride (Cl). The histograms of these ions are illustrated in Fig. 3; the highest frequency is 0–1000 mg/L and 0–2000 mg/L, respectively.

**Fig. 4:**
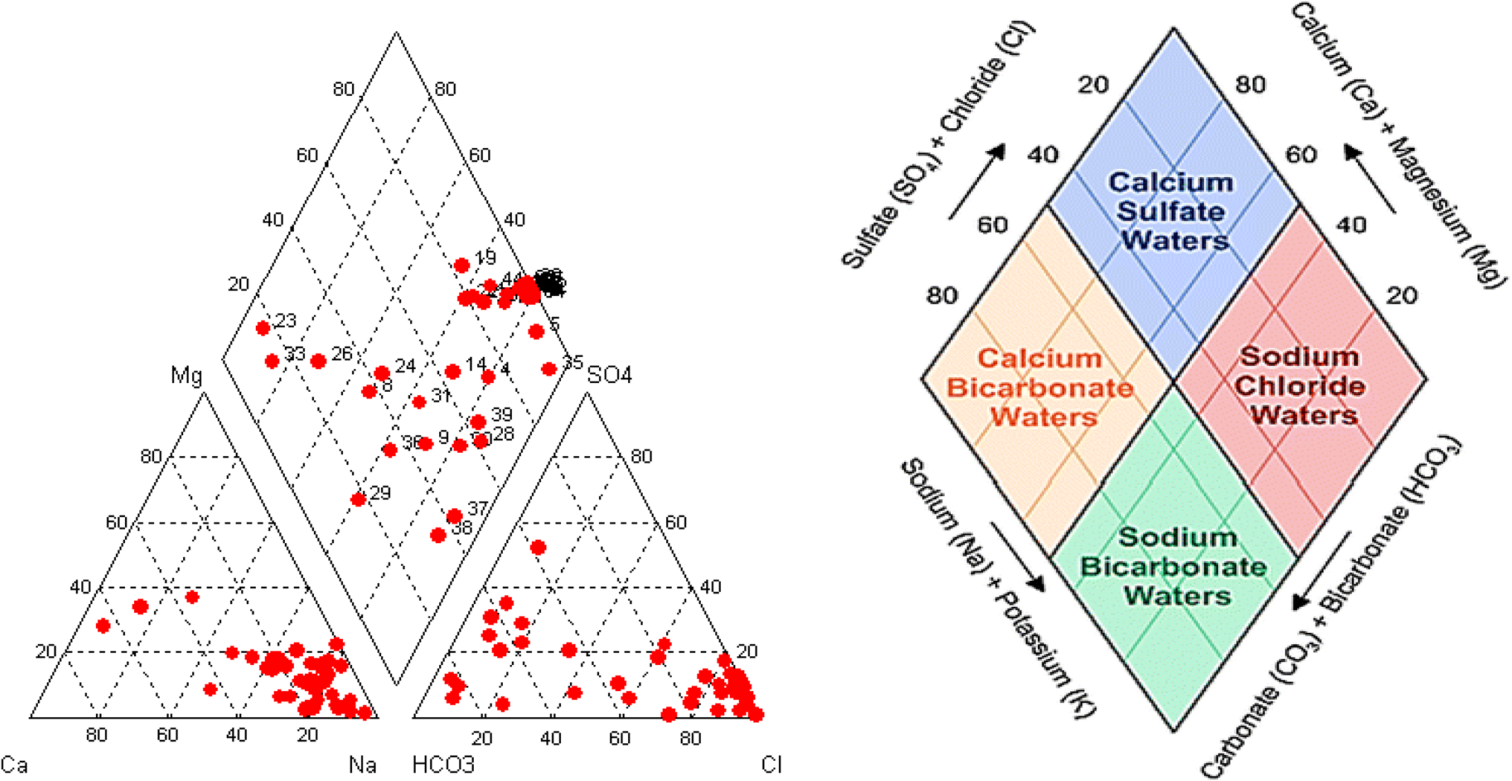
Piper diagram showing the hydrochemical types of thermal springs in Greece (the concentrations of ions are expressed in meq/L)

The general order of abundance of cations is: Na^+^ > Ca^2+^ > Mg^2+^ and of anions: Cl^−^ > SO_4_^2−^ > HCO_3_^−^ or Cl^−^ > HCO_3_^−^ > SO_4_^2−^. According to the major ionic composition and as it is revealed from the Piper diagram (Fig. 4), the predominant water type of spring waters is Na-Cl, especially in islands, can be associated with mixing phenomena between sea- and rainwater (Voudouris 2019; Papachristou et al. 2014). The hydrochemical type of Na-HCO_3_ is typical of springs located in the internal areas of North Greece (Lambrakis and Kallergis 2005).

The Na/Cl ion ratio (in meq/L) ranges between 0.73 and 10.25 (mean value 2.13) indicating the impacts on spring-water chemistry of seawater intrusion (Na/Cl < 1) in coastal areas and dissolution of minerals (Na/Cl > 1). In addition, the SO_4_/Cl ratio (in meq/L) ranges between 0.01 and 5.38 (mean value 0.70). Values less than 0.2 correspond to chloride water and values greater than 5 correspond to sulfate water affected by pyrite oxidation.

The presence of trace elements is related to the dissolution of minerals of the volcanic rocks and hydrothermal liquids. In a small number of thermal springs, e.g., Krinides, code number (c.n.) 23, a combination of mud therapy and hydrotherapy is offered, as mud has a beneficial and therapeutic effect on the human body, due to the presence of minerals (kaolinite, illite, halite, albite, calcite, chlorite, muscovite, etc.) and trace elements (Ni, Zn, Cu, Cr, U, Sb, V, Co, As, etc.) (Aggelidis 1990; Athanassoulis et al. 2016). The baths of Amarantos (c.n. 10) in NW Greece operate only with a natural flow of hot air (33–38 °C) coming from the inside of the earth (Bali 2016).

The maximum values of trace elements were recorded in the deepest aquifers characterized by long residence times for the groundwater (Papić 2016). Finally, the most radioactive spring-waters are those of Ikaria (mean value 3340 Bq/L), in terms of radon (Rn) concentration and Edipsos (mean value 2.5 Bq/L), in terms of radium (Ra) concentration.

## History of hydrotherapy by hydrothermal water and therapeutic effects

The use of water for various treatments (hydrotherapy) has been known since ancient times and is probably as old as mankind. Hydrotherapy (also called *aquatic therapy* or *balneotherapy* or *thermalism*) is one of the basic methods of treatment widely used in the system of natural medicine (Mooventhan and Nivethitha 2014).

As mentioned above, it is well established that at least during the Homeric era (ninth century BC), warm baths were used for body cleanliness, hygiene, and medical services. There is a chronological gap between the Homeric era and the founding of the first *Asclepieia,* with no available data. *Asclepieia* were sanctuaries and the first ancient places, where the water in combination with suitable climate conditions, played a very important role. In almost all the cities of the ancient Greek world, *Asclepieia* were operated by priests in the historical period, where people came for healing. The most well-known *Asclepieia* are *Asclepieion* of Epidaurus, *Asclepieion* of ancient Trikis (Trikala), *Asclepieion* of Kos Island, *Asclepieion* of Korinthos, *Asclepieion* of Athens, etc. It should be noted that *Asclepieia* continued to operate even after the predominance of Christianity until the end of the fifth century AD, with a continuous supply of therapy and health care for more than a millennium.

During the historical times, Hippocrates from the Aegean island of Kos (460–375 BC), the father of medicine and hydrotherapy, classified the natural waters into different types (meteoric, spring-water, etc.) and supported the diagnosis of diseases (Yapijakis 2009). He also suggested that some diseases relate to the bad water quality, e.g., brackish and acidic waters. He also recommended that seawater bath (thalassotherapy) and bathing in thermal waters could be beneficial in healing skin illnesses (Sazakli et al. 2016).

During the Hellenistic period (323–30 BC), a medical school was founded in Alexandria (Egypt), and the important role of water in hydrotherapy, as well as the water quality in health care, was widely recognized (Leoni et al. 2018). The therapeutic use of water has been recorded in ancient Egypt and Greece. Also, it was used by Roman and Egyptian royalty bathed with essential oils and flowers, while Romans had communal public baths for their citizens (Suppes et al. 2014; Schets et al. 2011; Dorevitch et al. 2011). Asclepiades of Bithynia (124–40 BC), the father of molecular medicine and founder of the methodic medicine in Rome, extensively favored hot and cold baths and successfully instigated a tradition that lasted for many centuries (Van Tubergen and Van der Linden 2002; Yapijakis 2009). Besides, other civilizations, e.g., China and Japan, had a long history of hydrotherapy before the Roman *thermae*. In Japan, the hydrotherapy took place primarily around hot springs or *onsen* (Angelakis et al. 2020a).

Hydrotherapy is a non-invasive and beneficial treatment for many patients, like patients with chronic diseases, disabilities, or trauma. Very often, those patients have a long history of medical treatments, including antibiotic treatments (Metcalfe 1877, 1898). Emerging and increasing antibiotic microbial resistance (AMR) represent one major threat to human health in Europe and worldwide (Leoni et al. 2018). Although pool water is usually disinfected, infections are known to occur either due to deficiencies in water treatment (Dufour et al. 2007). Therapies performed in a swimming pool cause a large release of bacteria. Bathers transfer approximately 105–106 CFU (colony-forming units) per person in 15 min to the surrounding water body (Suppes et al. 2016). Pseudomonas aeruginosa has been commonly isolated from the pool environment (Dorevitch et al. 2011).

Mooventhan and Nivethitha (2014) suggest that the use of water in various forms and in various temperatures can produce different effects on different parts of the body. In other words, they suggest that the hydrotherapy has a scientific evidence-based effect on various systems of the human body. Cacciapuoti et al. (2020) present the benefits of thermal water in chronic skin diseases, as well as the potential role of balneotherapy either alone or as a complement to conventional medical treatments.

Modern hydrotherapy includes: inhaling therapy (insertion of therapeutic substances in the form of gas), drinking therapy or positherapy (insertion of substances in the form of dissolved elements via drinking), balneotherapy (immersion of any part of the body in thermal water for therapeutic purposes), and mud therapy (use of mud with suitable chemical properties to cover part of the body). The following are detailed data on hydrotherapy in typical periods of Greek history.

### In prehistoric times

There is no relevant information on the development of water management and its use for therapeutic implications from prehistoric Greece. Chavara-Karahaliou (1984) in her Ph.D. thesis suggests that there was a healing shrine in the acropolis of Titani (close the ancient city Sykion, modern Kiato Korinthia), which was probably the first health center in the entire Hellenic world before the establishment of *Asclepieia*. The area, 15 km from the sea, is characterized by high sunshine, a small temperature range, low wind speeds, and low cloud cover and humidity, which are ideal and beneficial to human health. The historian Pausanias wrote (second century AD) that there was a statue of the goddess Hygieia (daughter of Asclepius), denoting her worship in this place. For the operation of this health center, spring waters of the wider area were used. In general, the ancient Greeks chose to establish health centers in the driest areas as they considered the dry climate as more convenient or healthier (Koutsoyiannis et al. 2008).

**Fig. 5:**
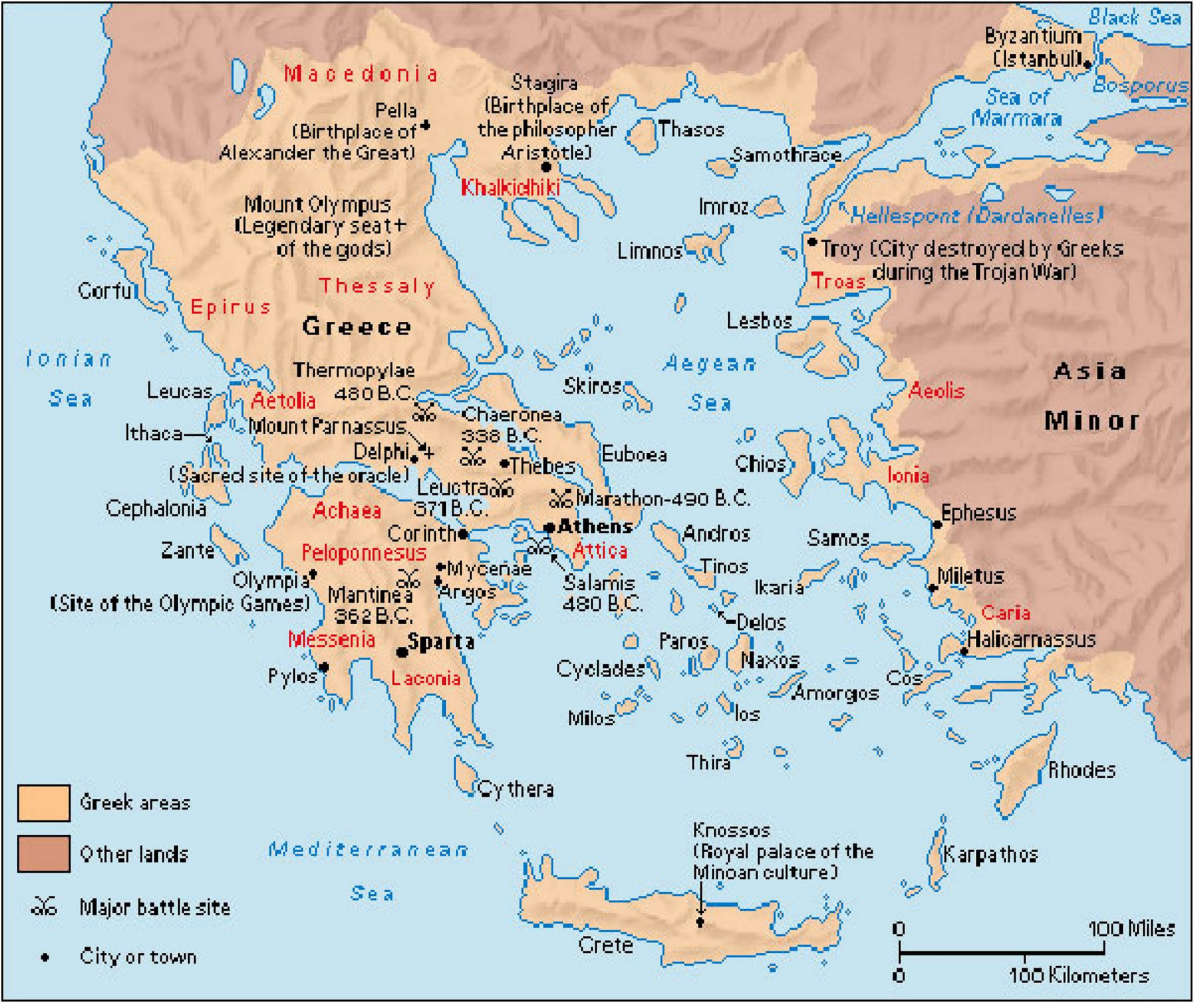
Map of ancient Greece showing the location of major ancient Greek centers (Krasilnikoff and Angelakis 2019)

Hydrotherapy was to some extent combined with safe and healthy water supply technologies. In Greece, where urban centers were located in relatively arid areas, water supply was an important factor in conservation and development. In many cases, the water supply of such settlements was based on aqueduct technologies, because Greeks preferred baths in fresh water from natural resources, including sea bathing (thalassotherapy). In addition to aqueducts, sedimentation tanks, sand filters, and other very useful devices for cleanings, purposes were used (Angelakis et al. 2020b). A map of ancient Greece showing the location of major ancient Greek centers is illustrated in Fig. 5.

### In historic times

Quite early in Classical times transferred medical powers from Mount Olympus down to earth and we have the appearance of Asclepius. Water appears to be a very important element of the *Asclepieia*, which are considered the first known places for therapy. More than 400 *Asclepieia* were operating in the ancient world, offering their valuable hydrotherapy services to the citizens. Data reported by Angelakis et al. (2020b) that waters from springs in *Asclepieia* had healing properties, offering its relief to sufferers and patients.

One representative case was the *Asclepieion* of Epidaurus, located in NE Peloponnesus, an area with mild climate and thermal springs. It was the most important therapeutic center in Greece. The purification of water supply was a key element of healing, which was based on periodic sleeping (*ὲγκοίμησις* in Greek) near water, as an imitation of the way in which the divine forces ensured their renewal, returning with periodic death to the earth from which they were born again (Angelakis et al. 2020a).

The history of mineral springs for therapeutic use begins in ancient Greece. The first observer of these springs with curative properties was the historian Herodotus (fifth century BC). He described certain curative springs and recommended spa therapy to be undertaken at particular seasons of the year. Hippocrates from Kos Island (460–375 BC) is considered the founder of medical science and the father of hydrotherapy (Angelakis et al. 2020a). He paid great attention to the different natural waters, which are of meteoric origin (rain, snow) or discharge from rocks (springs). These waters, namely mineral waters containing Fe, Cu, Ag, Zn, and other mineral elements. The mineral waters have therapeutic properties, due to the included tracer elements and other physicochemical properties. A little earlier (after ca 500 BC), Hippocrates discovered the healing powers of water and invented the practice of sieving water, and obtained the first bag filter, which was called the ‘Hippocratic sleeve’. The main purpose of the bag was to trap sediments that caused bad tastes or odors (Angelakis et al. 2020b).

During the Hellenistic period, the important role of water in health care and hydrotherapy was widely recognized. At the same time, the importance of cleanliness was also started to be recognized (Angelakis et al. 2020b). The Greek cities in the vast Hellenized empires had temples, theaters, libraries, gymnasia, and baths that often combined education with intensive physical exercise (Van Tubergen and Van der Linden 2002). Thermal baths at *Asclepieia* were popular combining worship and medical treatment by water submission to afflicted body parts or water immersion of the whole body in case of rheumatic and urogenital disorders (Van Tubergen and Van der Linden 2002; Lucore and Trümper 2013).

In all *Asclepieia*, the role of water and its cleanliness was a vital issue; it is pointed out that there was not *Asclepieion* without a source of water, mainly from springs. Very useful devices for cleanings purposes were used (Angelakis et al. 2020b). For example, the use of ceramic filters for water treatment, collected in cisterns, before its use in the *Asclepieion* of Emporiae (Hellenistic city of NW Spain, ca third century BC).

### In Roman, Byzantine, and Ottoman period

Ιn the Roman and Byzantine times, many doctors studied hydrotherapy and curative spa therapy. During the Middle Ages, with the human migration, the operation of baths declined, while many of them became obsolete. Due to sophisticated works, e.g., aqueducts, tanks, etc., improved bathing systems, named *thermae* (θέρμες in Greece), were developed during the Roman period. Archaeological finds from Roman baths are preserved in Athens, Thessaloniki, Traianoupoli, Philippi, Lesvos, Olympia, Thermi, etc. (Lucore and Trümper 2013). Asclepiades of Bithynia (124–40 BC), a Greek physician who introduced Greek medicine and Epicurean philanthropy in Rome, introduced treatments of hot and cold hydrotherapy instigating a tradition that lasted for many centuries (Yapijakis 2009). Asclepiades advocated the use of clean running water and avoidance of stagnant water in which “invisible tiny animals” (microbes) could cause disease if were inhaled (Yapijakis 2009, 2017). He recommended bathing for both therapeutic and preventative purposes for a number of conditions, as Galen (Galinos in Greek, 131–201 AD) wrote several centuries later (Van Tubergen and Van der Linden 2002). Eventually, three different types of baths existed in Roman times: home baths (*balnea*), private baths (balnea privata), and huge public baths (balnea publica) supported by water from aqueducts that could serve several hundreds of people (Van Tubergen and Van der Linden 2002; Lucore and Trümper 2013). The Minoan technologies for cleaning water supply were further developed during the Classical, Hellenistic, and Roman periods in Crete and other places of continental Greece (De Feo et al. 2013).

**Fig. 6:**
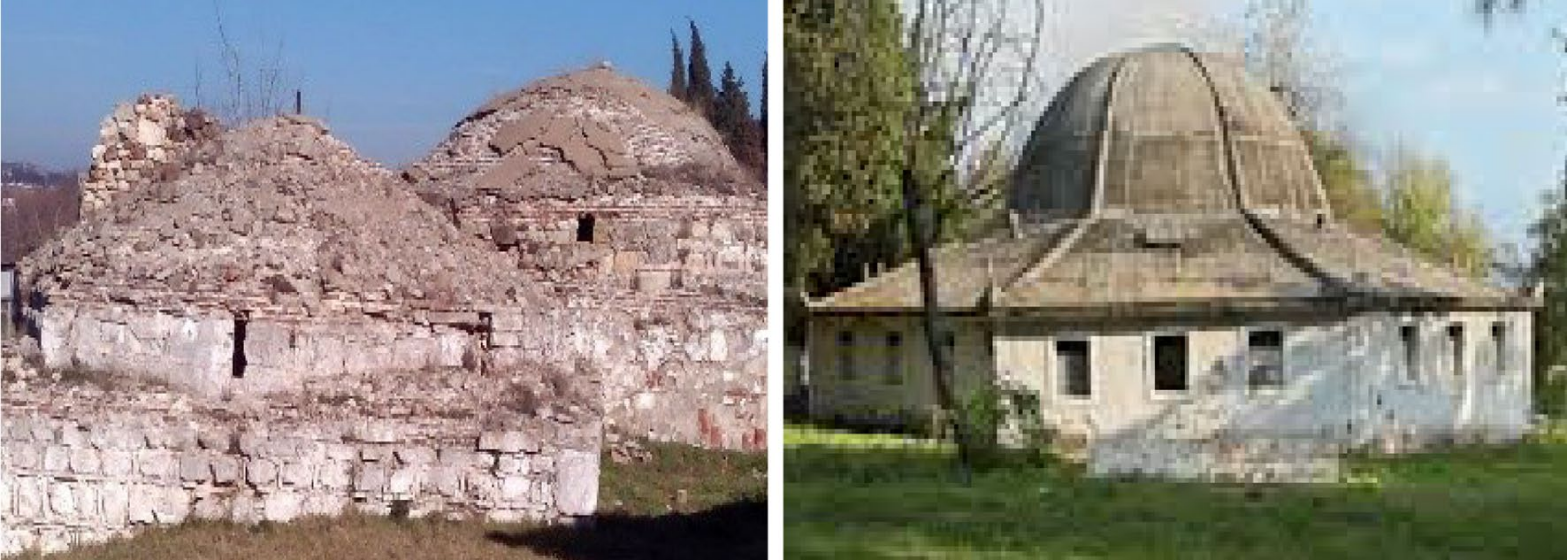
Left: Baths of Τraianoupolis (Ottoman period). Right: Baths of Thermi, Thessaloniki

**Fig. 7:**
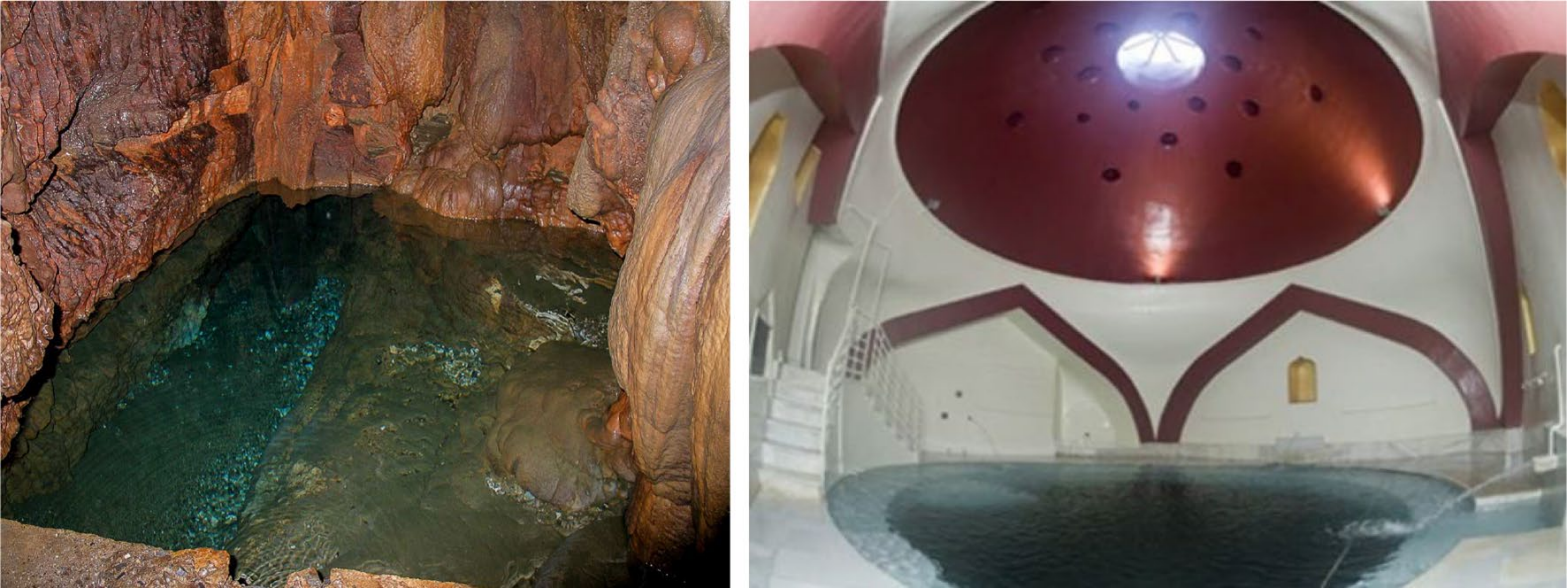
Left: Spring of Cave in Ikaria Island, Right: Baths of Lagadas, Thessaloniki

Representative baths of this period are the baths of Τraianoupolis (NE Greece, code number 43), a city founded by the emperor Marcus Ulpius Traianus and flourished in the second century AD. On the east side, there are four vaulted buildings with public baths; two of them of the Ottoman period (Fig. 6) and the others two of the Roman period in the primary phase with later additions and restorations. It is impressive that the hot spring-water was used for the heating of a hostel (*Chana*), where people stayed close to the baths, through a ceramic pipe. This is considered one of the first applications of geothermal energy (Kyrkoudis 1992). Another case is the baths of Thermi (c.n. 35), located 20 km to the east of Thessaloniki. Their acid salt waters are suitable for rheumatisms, ailments of the vascular system, etc. Today, they do not operate and are in the process of change of ownership and renovation (Fig. 6). Lagadas baths (c.n. 24), located at a distance of 19 km of Thessaloniki, operate as an organized mineral spring ever since antiquity. Today the spa of Lagadas developed on the site of the byzantine installations including private and public baths (Fig. 7).

The last case is a super-hot thermal aquifer system developed in the Therma basin of Ikaria Island (c.n. 2,3) including springs (35–52 °C), which are characterized by the presence of radon (Ra). The hydrochemical type of water is Na-Cl. Baths were built during the Hellenistic period, which were completed during the Roman period. The new spa town was developed and operates near the ancient Therma (Fig. 7).

During the Byzantine period and the rise of Christianity, due to religious beliefs, hydrotherapy was gradually reduced and eventually abandoned. The use of public baths ceased during the 6–7 century AD. Many thermal or mineral springs were characterized as holy springs or holy water (αγίασμα in Greek). However, some doctors of this period stressed the importance of hydrotherapy, e.g., Orivasios. Therefore, the use of baths was limited to the public baths (*Hamam*) of the Islamic world and hydrotherapy reappeared in eighteenth century in Europe (Aggelidis 2008).

## Modern times

**Fig. 8:**
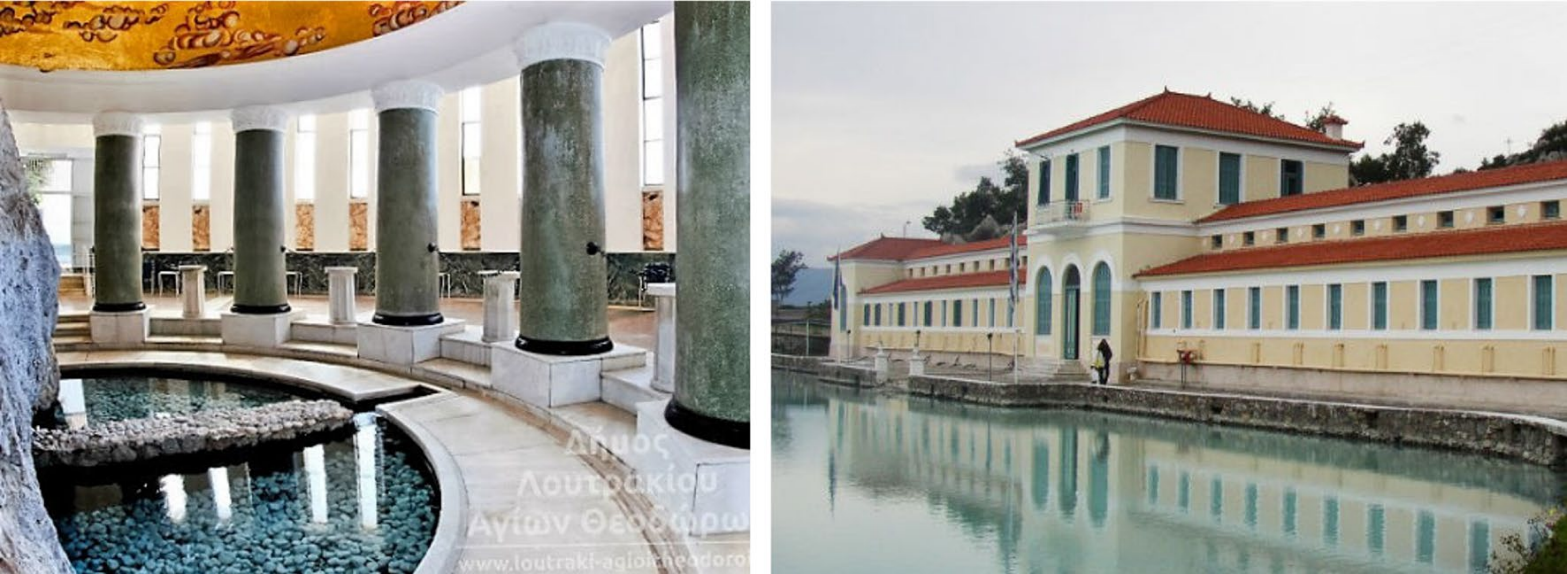
Left: Municipal thermal spring of Loutraki Korinthia (http://new.loutraki-agioitheodoroi.gr). Right: Methana thermal springs

In modern times, the *thermalism* in Greece begins in the era of the Prime Minister Ioannis Kapodistrias (1776–1831). At that times, thermal spas refer to the thermal waters of hot natural springs. In modern times, the interest for the recording, studying, and developing of the curative springs began from Kapodistrias Government. The first spa towns with modern thermal baths were Kythnos, Methana (Fig. 8), Loutraki in Corinth, Edipsos, etc. The first baths were these of Kythnos Island and constructed by marble bathtubs in 1845. In 1877, the curative baths of Edipsos started being reused and the thermal city of Evia became a major attraction for tourists. Later, after the Second World War, many spa towns developed. A modern paradigm is the Loutraki rehabilitation center located in the Korinthian Gulf (Fig. 8). It offers treatments for neurological, musculoskeletal disorders to name a few, following a holistic treatment approach of the patient.

The Hellenic Association of Municipalities with Thermal Springs (H.A.M.T.S), founded in 1983, supports members for the protection and development of thermal natural resources. Ministry of Tourism is responsible for the recognition-certification of springs.

In Greece since the ancient times until today, people take care of their health with the use of the therapeutic natural springs. The dominant idea of Hippocrates that the therapeutic treatment must take place in a pleasant and healthy environment remains unchanged through the ages. The bathing places of Greece remained, throughout time, places of meetings, recreation, and treatment (Coveou 2017).

The spa towns were mainly used until the period of 1970s as the new middle age people began to use these during their vacation time, taking advantage of their proximity to the sea beaches. That way the spas towns were no longer high-priority used places and were only used incidentally by those preferring cosmopolitan beaches. Thus, they gradually came to be used only by the older people, and their use came to be used primarily for therapeutic reasons (Kouskoukis 2013). In this way, spas came to be regarded as destinations for the old people for the therapy their illnesses (Appendix).

## Future trends

Over the past decades, a re-assessment of the use of mineral water for the treatment of several diseases has investigated. Furthermore, water therapy is being applied in many countries of the world that have a variety of mineral springs considerably different in their hydrogeologic origin, temperature, and chemical composition (Cacciapuoti et al. 2020). It is pointed out that medical tourism has become one of the fastest-growing forms of tourism on a global scale. Therapeutic treatments in different forms: hydrotherapy (balneotherapy), inhalation, and thalassotherapy are not only for the elderly but for everyone who needs rehabilitation, prevention, treatment, and wellness.

Thermal water offers several advantages: there is no need of using chemicals or potentially harmful drugs; there are almost no side effects during and after treatment, and there is a low risk to the patient's general health and well-being (Cacciapuoti et al. 2020). Nowadays, with the new trends in holistic medicine, traditional spa therapy is a kind of spa tourism aiming at not only at patients but at healthy people who wish to combine their vacations with preventive services, revitalization, and relaxation.

From its establishment in 1952, the Hellenic Organization of Tourism (EOT) promotes and supports the growth of tourism, as regard to refers to thermal–mineral springs. The new law 17,414/Government Newspaper 2215/2-10-2009 and the amended law 22,527/Government newspaper 2997/6-11-2014 define the procedures for the recognition of natural thermal resources. Greece can play a leading role as it has excellent climate conditions and an extraordinary number of thermal springs covering a wide range of therapies. Greek spa resorts can be developed and evolved into health tourism centers, a kind of treatment center that also offers recreational and leisure activities. Because many of them are close to the sea can be combined the hydrotherapy with seawater therapy, as well as with the Mediterranean diet and athletic activities.

It is pointed out that in the post-pandemic period, some spas offer beauty treatments and high-level medical programs, called medspas (medical spas). MedSpas have climbed the ranking of places dedicated to well-being and vacation destinations around the world, combining care programs and beauty treatments.

## Discussion and conclusions

Hydrotherapy (*balneotherapy*), the therapeutic use of water, has been known since ancient times and is considered that it has a scientific evidence-based effect on different parts of the human body. Warm baths were used for body cleanliness, hygiene, and medical services, since the Homeric era (ninth century BC). During the Classical period, water appears to be a very important element of the *Asclepieia*, which were located where clean water was available, and are considered the first known therapy centers. Spring-waters in *Asclepieia* have healing properties, offering wellness, and therapy to patients.

During the Hellenistic times, the important role of water in health was widely recognized. In the Roman era, many doctors following the Hippocratic medicine involved hydrotherapy in the treatment, using baths (*thermae)*. During the Middle Ages, with the human migration, the operation of baths declined. During the Ottoman period, public baths (well known as *Hamam*) close to thermal springs were used until the modern era.

Nowadays, water therapy is being applied in many countries of the world that have different types of mineral springs, e.g., in Europe, there are about 1400 centers of thermal medicine that operate in the form of health resorts. Many springs are recorded in Greece, some of them are characterized as thermo-minerals, due to the high temperature (> 20 °C) and the high concentration of chemical elements and tracers. The thermal springs of Greece are correlated with its geographical position between Eurasia and Africa, the volcanic activity, the geological–hydrogeological regime, and the presence of tectonic faults. Therefore, the geotectonic regime of Greece favors the circulation of geothermal fluids and the development of springs. The exploitation of geothermal fields has to be further increased but only in a sustainable way.

According to the Hellenic Survey of Geology and Mineral Exploration, there are 750 thermal springs in Greece; 128 of them are active, and 52 are recognized and certified by the Ministry of Tourism. Most thermal springs are recorded in Central and Northern Greece.

The temperature of springs ranges between 20.5 and 82.3 °C; the highest value is recorded in Lesvos Island. Besides, high temperatures in spring-water are recorded in Aegean active volcanic arc. The thermal waters show an alkaline behavior, as pH values are greater than 7.0. The predominant type of spring waters is Na-Cl, especially in islands, can be related to mixing phenomena between seawater and rainwater. The hydrochemical type of Na-HCO_3_ is recorded in springs located in the internal post-orogenic basins (Mygdonia, Strymonas basin, etc.) of North Greece (Lambrakis and Kallergis 2005). The presence of trace elements is associated with the dissolution of minerals of the volcanic rocks and hydrothermal liquids. The majority of thermal springs have low content of CO_2_ and H_2_S, the main gases of geothermal fluids.

The main therapeutic effects of the thermal springs refer to rheumatism, skin (derma) diseases, gynecological diseases, and mainly the rejuvenation of the human body. Furthermore, rehabilitation clinics linked to geothermal springs offer treatments for neurological, musculoskeletal, and multi-systemic disorders and for improvement of permanent disabilities.

Nowadays, according to the new trends in holistic medicine, traditional hydrotherapy is a kind of spa tourism aiming at not only at patients but at healthy people who wish to combine their holidays with revitalization, and relaxation. Greece can play a leading role in this sector as it has excellent climate conditions and an extraordinary number of thermal springs covering a wide range of therapies combining hydrotherapy with seawater therapy, as well as with the Mediterranean diet and athletic activities. It is pointed out that spa tourism constitutes a significant pillar for economic development in Greece, especially in islands, e.g., Lesvos, Ikaria (Lambrakis and Stamatis 2008). In addition, thermal tourism could be combined with winter programs and other forms of alternative tourism (Nikoli and Lazakidou 2019).

According to the H.A.M.T.S. association, in 2015, approximately 882,500 visits were made to public and private thermal baths. It should be noted that the maximum number of visitors was recorded in 2009 (approximately 2,280,000). The economic crisis has led to a significant reduction. Given that Greece welcomed about 29 million tourists (2019), it is concluded that a small percentage of them visit the thermal baths, while the larger percentage prefers the “sun-sea-sand” touristic model (Nikoli and Lazakidou 2019). Public municipal thermal baths received more visitors (68%) than their private counterparts. It should be noted that the prolonged financial crisis in Greece has resulted in a significant reduction in the number of visitors, as well as the number of days they spent in the thermal springs. Although there are no available data, the pandemic crisis of COVID-19 significantly reduced the number of bath visits, as they remained closed for a long time.

Greek tourism policy should provide incentives and financial support to the thermal centers to improve their facilities and offered services. In addition, it is very important that health systems should provide subsidies to visitors, especially for the most financially and socially weak. It is underlined that World Health Organization (WHO 2016) has defined guidelines for safe recreational water environments, e.g., swimming pools and similar environments. Today, many people seek treatment and recreation at thermal springs. Thus, the return of medicine to the natural ways of treatment and the acceptance of the psychosomatic treatment are observed, in which the development of the spas contributes as it happened in ancient Greece.

## Data Availability

The results of this study are freely available.

## Appendix

**Table:** Thermal springs for hydrotherapy in Greece certified by the Ministry of Tourism (Data provided by H.A.M.T.S. and H.S.G.M.E. with modification)

	Thermal springs	*T* (°C)	Characteristics of thermal springs	Diseases
1	Adamandas, Milos	38.8	Thermal spring of iron, sulfur, and chloride sodium	Rheumatic, gynecological, neurological
2	Therma Apollona, Ikaria	41.7	Saline, spring mainly of sodium and chloride	Rheumatic, gynecological, neurological
3	Agia Kyriaki, Ikaria	34.9	Radioactive saline spring	Rheumatic, gynecological, neurological
4	Agia Paraskevi, Chalkidiki	22.9	Alkaline of chloride and sodium and alkaline earth acid springs	Rheumatic, gynecological, skin, urinary
5	Agiasmata, Chios	55.8	Ηyperthermic spring of chloride and sodium, weakly radioactive	Rheumatic, gynecological, skin, neurological
6	Agioi Anargiroi, Kythnos	20.4	Saline spring of iodine and bromide	Rheumatic, gynecological, skin, neurological, urinary
7	Agios Nikolaos, Αttica	32.6	Thermal spring of chloride and sodium	Rheumatic, gynecological, neurological
8	Agkistro, Serres	37.6	Alkaline springs	Rheumatic
9	Agrapidia-Limnohori, Florina	25.2	Hypothermic spring mainly of sodium and sulfur, weakly radioactive	Rheumatic, skin, gastrointestinal
10	Amarantos, Ioannina	39.0	Radioactive spring of iron	Rheumatic, neurological, respiratory
11	Edipsos, Evia	65.8	Saline, hyperthermic springs mainly of chloride and sodium of which the 4 springs are radioactive	Rheumatic, gynecological, skin, neurological
12	Asklipios, Ikaria	41.8	Radioactive and saline springs	Rheumatic, gynecological, neurological
13	Eftalou, Lesvos	45.8	Radioactive springs of iron	Rheumatic, gynecological, skin, neurological
14	Eleftheres, Kavala	41.0	Alkaline spring of chloride and sodium, alkaline earth acid springs	Rheumatic, skin
15	Kaiafas, Ilia	28.5	Thermal spring of hydrochloride with a large amount of free hydrogen sulfide	Rheumatic, skin
16	Kakavas, Kythnos	50.9	Saline spring mainly of iron, iodine and bromide	Rheumatic, gynecological, skin, neurological, urinary
17	Kallidromos, Fthiotida	37.5	Ηyperthermic radioactive spring of chloride, and sometimes as hydrochloride- hydrogen sulfide	Rheumatic, gyneological, skin
18	Kamena Vourla Fthiotida	35.2	Radioactive springs (2 thermal and 2 hypothermic) and hydrochloride	Rheumatic, gynecological, skin, urinary
19	Kavasila and Pixaria, Ioannina	30.6	Sulfur hyporthermic spring	Rheumatic, gynecological, skin, neurological
20	Kimolos	51.9	Spring mainly of sodium, calcium, magnesium, radium, iron, iodine, phosphorus, and sulfur	-
21	Kolpou Geras, Lesvos	34.7	Thermal spring mainly of chloride and sodium	Rheumatic, neurological
22	Kremasta Valtou, Etolokarnania	28.9	Sulfur hypothermic spring	-
23	Krinides, Kavala	44.6	Hypothermic spring mainly of calcium and magnesium	Rheumatic, gynecological, skin, neurological, urinary, gastrointestinal
24	Lagadas, Thessaloniki	35.9	Hypothermic spring mainly of chloride, sodium, potassium and calcium	Rheumatic, gynecological, skin, urinary
25	Loutraki, Korinthia	30.4	Radioactive spring	Rheumatic, skin, urinary, gastrointestinal
26	Loutrohori, Pella	21.9	Hypothermic spring mainly of sulfide, sodium, calcium and potassium	Rheumatic, skin, respiratory, gastrointestinal
27	Mandraki, Nisiros	44.9	Saline spring	Rheumatic, gynecological
28	Nea Apollonia, Thessaloniki	46.8	Alkaline sulfur springs	Rheumatic, gynecological, skin, neurological, respiratory
29	Nigritas (Thermes), Serres	36.6	Alkaline acid spring of alkaline earth	Rheumatic, gynecological, skin, gastrointestinal
30	Palaiovraha, Fthiotida	22.9	Hypothermic alkaline spring of sulfur	Rheumatic, skin, respiratory
31	Platystomo, Fthiotida	31.9	Hypothermic alkaline spring of sulfur, weakly radioactive	Rheumatic, gynecological, skin, urinary, gastrointestinal
32	Polichnitos, Lesvos	82.3	Most thermal springs of chloride and sodium	Rheumatic, gynecological, skin, neurological
33	Pozar, Loutraki Pella	35.4	Equal thermal spring mainly of calcium, potassium, magnesium and boron, weakly radioactive	Rheumatic, gynecological, skin, neurological
34	Psarotherma,Evros	43.5	Hydrochloride saline, weakly radioactive	Rheumatic, gynecological, skin
35	Sedes-Thermi, Thessaloniki	37,4	Acid salt waters	Rheumatic, gynecological, skin, neurological
36	Sidirokastro, Serres	42.9	Alkaline earth springs	Rheumatic, gynecological, neurological
37	Smokovo, Karditsa	41.7	Alkaline sulfur springs	Rheumatic, gynecological, skin, respiratory, urinary, gastrointestinal
38	Soulanta, Karditsa	40,3	Hyperthermic spring mainly of sodium and fluorine, weakly radioactive	Rheumatic, skin
39	Therma, Limnos	38.8	Hyperthermic spring of sodium and chloride	Rheumatic
40	Therma, Kalymnos	35.5	Saline spring	Rheumatic
41	Therma Agios Fokas, Kos	45.0	Saline spring	Rheumatic, skin, neurological, respiratory, gastrointestinal
42	Thermopiles, Fthiotida	41.1	Hyperthermic hydrochloride springs, weakly radioactive	Rheumatic, gynecological, neurological
43	Traianoupoli, Evros	51.6	Hyperthermic spring of sodium and chloride, radioactive	Rheumatic, gynecological, neurological, skin, respiratory, urinary, gastrointestinal
44	Ypati, Fthiotida	31.5	Spring of hydrogen sulfide and chloride, acid spring of alkaline earth, weakly radioactive	Skin, neurological, urinary, gastrointestinal

## References

[CR1] Aggelidis Z (1990) Mud occurences in Greece and possibilities of utilization in therapeutic tourism. In: Proc. of 2nd conference on thermometallic waters. Thessaloniki 7–9 October 1988, pp 74–87 (in Greek)

[CR2] Aggelidis Z (2008) Thermal natural resources and thermalism. Hellenic Ministry of Education and Religious Affairs (in Greek)

[CR3] Angelakis AN, Savvakis YM, Charalampakis G (2007). Aqueducts during the Minoan era. Water Sci Technol Water Supply.

[CR4] Angelakis AN, Antoniou G, Yapijakis C, Tchobanoglous G (2020). History of hygiene focusing on the crucial role of water in the Hellenic Asclepieia (i.e., Ancient Hospitals). Water.

[CR5] Angelakis AN, Voudouris KS, Tchobanoglous G (2020). Evolution of water supplies in the Hellenic World focusing on the water treatment and modern parallels. Water Supply.

[CR6] Athanassoulis C, Zaimis S, Chatziapostolou A, Agalaniotou S (2016) Therapeutic mud occurences in Greece: Mineralogical and geochemical composition of the Sagiada mud. Bulletin of the Geological Society of Greece, vol. XLVIII

[CR7] Bali E (2016) Classification of thermal springs based on hydrogeological, hydrochemical and geothermal criteria: Western and central Macedonia, Greece. MSc Thesis (K. Voudouris, supervisor), Dept. of Geology, Aristotle University of Thessaloniki

[CR8] Cacciapuoti S, Luciano MA, Megna M, Annunziata MC, Napolitano M, Patruno C, Scala E, Colicchio R, Pagliuca C, Salvatore P, Fabbrocini G (2020). The role of thermal water in chronic skin diseases management: a review of the literature. J Clin Med.

[CR9] Chaviara-Karahaliou S (1984) Asklepieon of ancient Titani: the first center of health in Greek land. PhD Thesis, University of Ioannina, Dept. of History of Medicine, p 85

[CR10] Coveou M (2017) Greece, a paradise of hot springs. https://www.greece-is.com/thermal-springs/

[CR11] Crouch D (2003). Water management in Ancient Greek cities.

[CR12] D’Alessandro W, Brusca L, Martelli M, Rizzo A, Kyriakopoulos K (2010) Geochemical characterization of natural gas manifestations in Greece. In: Proc of 12 Intern. Congress of the Geological Society of Greece, Patras, Greece, May 2010. Bulletin Geol. Soc. Greece 43:5, 2327–2337

[CR13] De Feo G, Angelakis AN, Antoniou GP, El-Gohary F, Haut B, Passchier CW, Zheng XY (2013). Historical and technical notes on aqueducts from Prehistoric to Medieval times. Water.

[CR14] Dorevitch S, Panthi S, Huang Y, Li H, Michalek AM, Pratap P, Wroblewski M, Liu L, Scheff PA, Li A (2011). Water ingestion during water recreation. Water Res.

[CR15] Dufour AP, Evans O, Behymer TD, Cantu R (2007). Water ingestion during swimming activities in a pool: a pilot study. J Water Health.

[CR16] Fytikas M (1978). Geothermal situation in Greece. Geothermics.

[CR17] Hellenic Association of Municipalities with Thermal Springs (H.A.M.T.S.), Thessaloniki, Greece, https://www.thermalsprings.gr/index.php/en/. Accessed 20 December, 2020

[CR18] Hellenic Survey of Geology and Mineral Exploration (H.S.G.M.E.) (2009) Periodic monitoring of thermal spring of Greece. Unpublished technical report. Athens

[CR19] Kallegia A (2000). Asclepieia, ancient health centers. Corpus.

[CR20] Kouskoukis Κ (2013) The future of thermal springs, http://www.kedke.gr/tourism/wpcontent/uploads/2013/09/ (in Greek) (accessed 28 April 2018)

[CR21] Koutsoyiannis D, Zarkadoulas N, Angelakis AN, Tchobanoglous G (2008). Urban water management in ancient Greece: legacies and lessons. ASCE J Water Resour Plann Manag.

[CR22] Krasilnikoff J, Angelakis AN (2019). Water management and its judicial contexts in ancient Greece: a review from the earliest times to the Roman period. Water Policy.

[CR23] Kyrkoudis Th (1992) History of Traianoupolis. Centre of Thracian studies. Komotini, pp 227–279 (in Greek)

[CR24] Lambrakis N, Kallergis G (2005). Contribution to the study of Greek thermal springs: hydrogeological and hydrochemical characteristics and origin of thermal waters. Hydrogeol J.

[CR25] Lambrakis N, Stamatis G (2008). Contribution to the study of thermal waters in Greece: chemical patterns and origin of thermal water in the thermal springs of Lesvos. Hydrol Process.

[CR26] Lekkas N (1938) The 750 metallic springs of Greece. Ministry of Finance, Issue of thermal springs, Athens, pp 249–275

[CR27] Leoni E, Katalani F, Marini S, Dallolio L (2018). Legionellosis associated with recreational waters: a systematic review of cases and outbreaks in swimming pools, spa pools, and similar environments. Int J Environ Res Public Health.

[CR28] Lucore SK, Trümper M (2013) Greek baths and bathing culture: new discoveries and approaches. Babesch Suppl. 23, Leuven

[CR29] Martin R, and Metzger H (1976) La religion grecque. Presses Universitaires de France. Coll. "Sup-L' Historien" 22, Paris, France, p. 208 (in French)

[CR30] Metcalfe R (1877) Sanitus sanitum et omnia sanitus. The Co-operative Printing Co, vol 1, London, UK. Retrieved 4 November 2009

[CR31] Metcalfe R (1898) Life of Vincent Priessnitz, founder of Hydropathy. Simpkin, Marshall, Hamilton, Kent & Co., Ltd, London, UK. Retrieved 3 December 2009. Full text at Internet Archive (archive.org)

[CR32] Mooventhan A, Nivethitha L (2014). Scientific evidence-based effects of hydrotherapy on various systems of the body. N Am J Med Sci.

[CR33] Nikoli G, Lazakidou A (2019). A review of thermal tourism in Europe and Greece. Tourism.

[CR34] Papachristou M, Voudouris K, Karakatsanis S, D’Alessandro W, Kyriakopoulos K (2014) Geological setting, geothermal conditions and hydrochemistry of south and southeastern Aegean geothermal systems. In: Baba A, Bundschuh J, Chandrasekharam D (eds) “Geothermal Systems and Energy Resources: Turkey and Greece”. CRC Press, Chapter 4, pp 47–75

[CR35] Papić P (ed.) (2016) Mineral and thermal waters of southeastern Europe. ISSN 2199–9155

[CR36] Platakis E (1966). Bibliography of the thermal springs of Greece. Arch Natl Univ Athens School Pharm.

[CR37] Risse GH (1999) Mending bodies, saving souls: a history of the hospitals. Oxford University Press, Inc., 198 Madison Avenue, New York, 10016 NY, USA

[CR38] Routh H, Bhowmik KR, Parish LC, Witkowski JA (1996). Balneology, mineral water, and spas in historical perspective. Clin Dermatol.

[CR39] Sazakli E, Sazaklie E, Leotsinidis M, Varnavas SP (2016) Hydrotherapy: An ancient art, legacy of Hippocrates, the father of Medicine. In: Proc of 4th IWA international symposium on “Water and Wastewater Technologies in Ancient Civilizations”, September 17–19, 2016, Coimbra, Portugal. http://www.wwac2016.com/

[CR40] Schets FM, Schijven JF, Husman AMD (2011). Exposure assessment for swimmers in bathing waters and swimming pools. Water Res.

[CR41] Suppes LM, Abrell L, Dufour AP, Reynolds KA (2014). Assessment of swimmer behaviors on pool water ingestion. J Water Health.

[CR42] Suppes LM, Canales RA, Gerba CP, Reynolds KA (2016). Cryptosporidium risk from swimming pool exposures. Int J Hyg Environ Health.

[CR43] van Tubergen A, van der Linden S (2002). A brief history of spa therapy. Ann Rheum Dis.

[CR44] Voudouris K, Angelakis AN, Mays LW, Koutsoyiannis D, Mamassis N (2012). Diachronic evolution of water supply in eastern Mediterranean. Evolution of water supply through the Millennia.

[CR45] Voudouris K (2019) Classification and codification of karst aquifers in Aegean islands. In: Proc International Earth Science Colloquium on the Aegean Region (IESCA). 7–11 October 2019, Dokuz Eylül University, Izmir, Turkey, pp 146–150

[CR46] Voudouris K, Valipour M, Kaiafa A, Zheng XY, Kumar R, Zanier K, Kolokytha E, Angelakis A (2019). Evolution of water wells focusing on Balkan and Asian civilizations. Water Supply.

[CR47] WHO (World Health Organization) (2016) Guidelines for safe recreational water environments, vol. 2: Swimming pool and similar environments. Available online (accessed on 29 December 2020): www.who.int/water_sanitation_health/bathing/srwe2full.pdf.

[CR48] Yapijakis C (2009). Hippocrates of Kos, the father of clinical medicine, and Asclepiades of Bithynia, the father of molecular medicine. In Vivo.

[CR49] Yapijakis C (2017) Ancestral Concepts of Human Genetics and Molecular Medicine in Epicurean Philosophy. In: Petermann H, Harper P, Doetz S (eds) History of human genetics. Springer

